# Supplementary material for: Congruence and Complementarity of Differential Mobility Spectrometry and NMR Spectroscopy for Plasma Lipidomics
Source: Metabolites. 2022 Oct 27;12(11):1030. doi: 10.3390/metabo12111030 (PMC9699282; doi:10.3390/metabo12111030)
Supplement: Supplementary file 1 [file metabolites-12-01030-s001.zip › LPIV_Supplemental Materials Final.pdf]

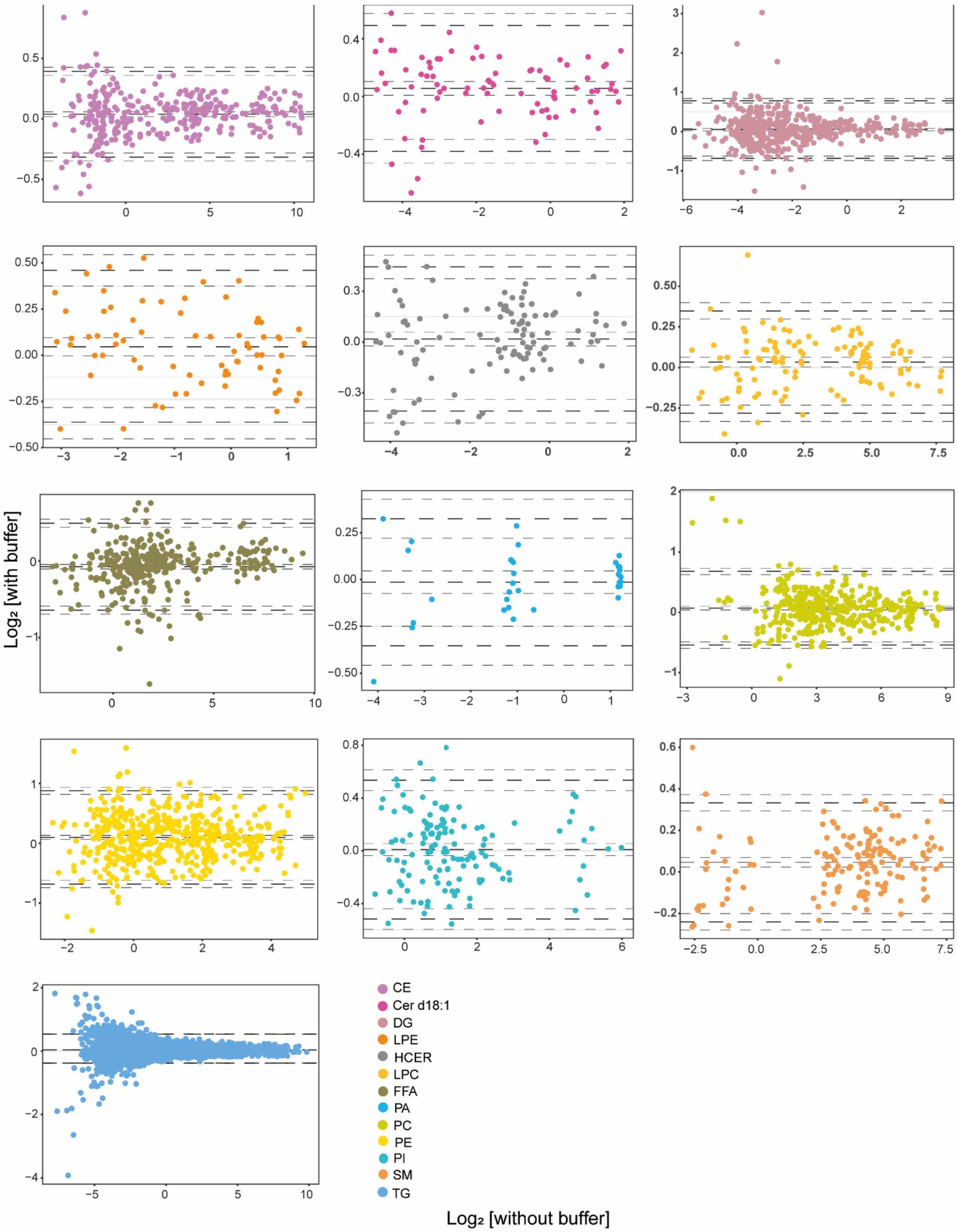

Supplemental Figure S1. Bland-Altman analysis of DMS-SLA measured samples with and without buffer addition.

Table S1: Abbreviations and full names of lipoprotein particles measured with NMR

Names	Abbreviations
Calculated Figures, Apo-A1 / Apo-B100, Apo-B100/Apo-A1	ABA1
Calculated Figures, IDL Particle Number, IDL Particle Number	IDPN
Calculated Figures, LDL Cholesterol / HDL Cholesterol, LDL-Chol/HDL-Chol	LDHD
Calculated Figures, LDL Particle Number, LDL Particle Number	LDPN
Calculated Figures, LDL-1 Particle Number, LDL-1 Particle Number	L1PN
Calculated Figures, LDL-2 Particle Number, LDL-2 Particle Number	L2PN
Calculated Figures, LDL-3 Particle Number, LDL-3 Particle Number	L3PN
Calculated Figures, LDL-4 Particle Number, LDL-4 Particle Number	L4PN
Calculated Figures, LDL-5 Particle Number, LDL-5 Particle Number	L5PN
Calculated Figures, LDL-6 Particle Number, LDL-6 Particle Number	L6PN
Calculated Figures, Total ApoB Particle Number, Total Particle Number	TBPN
Calculated Figures, VLDL Particle Number, VLDL Particle Number	VLPN
HDL Subfractions, Apo-A1, HDL-1	H1A1
HDL Subfractions, Apo-A1, HDL-2	H2A1
HDL Subfractions, Apo-A1, HDL-3	H3A1
HDL Subfractions, Apo-A1, HDL-4	H4A1
HDL Subfractions, Apo-A2, HDL-1	H1A2
HDL Subfractions, Apo-A2, HDL-2	H2A2
HDL Subfractions, Apo-A2, HDL-3	H3A2
HDL Subfractions, Apo-A2, HDL-4	H4A2
HDL Subfractions, Cholesterol, HDL-1	H1CH
HDL Subfractions, Cholesterol, HDL-2	H2CH
HDL Subfractions, Cholesterol, HDL-3	H3CH
HDL Subfractions, Cholesterol, HDL-4	H4CH
HDL Subfractions, Free Cholesterol, HDL-1	H1FC
HDL Subfractions, Free Cholesterol, HDL-2	H2FC
HDL Subfractions, Free Cholesterol, HDL-3	H3FC
HDL Subfractions, Free Cholesterol, HDL-4	H4FC
HDL Subfractions, Phospholipids, HDL-1	H1PL
HDL Subfractions, Phospholipids, HDL-2	H2PL
HDL Subfractions, Phospholipids, HDL-3	H3PL
HDL Subfractions, Phospholipids, HDL-4	H4PL
HDL Subfractions, Triglycerides, HDL-1	H1TG
HDL Subfractions, Triglycerides, HDL-2	H2TG
HDL Subfractions, Triglycerides, HDL-3	H3TG
HDL Subfractions, Triglycerides, HDL-4	H4TG
LDL Subfractions, Apo-B, LDL-1	L1AB
LDL Subfractions, Apo-B, LDL-2	L2AB
LDL Subfractions, Apo-B, LDL-3	L3AB
LDL Subfractions, Apo-B, LDL-4	L4AB
LDL Subfractions, Apo-B, LDL-5	L5AB
LDL Subfractions, Apo-B, LDL-6	L6AB
LDL Subfractions, Cholesterol, LDL-1	L1CH
LDL Subfractions, Cholesterol, LDL-2	L2CH
LDL Subfractions, Cholesterol, LDL-3	L3CH
LDL Subfractions, Cholesterol, LDL-4	L4CH

LDL Subfractions, Cholesterol, LDL-5	L5CH
LDL Subfractions, Cholesterol, LDL-6	L6CH
LDL Subfractions, Free Cholesterol, LDL-1	L1FC
LDL Subfractions, Free Cholesterol, LDL-2	L2FC
LDL Subfractions, Free Cholesterol, LDL-3	L3FC
LDL Subfractions, Free Cholesterol, LDL-4	L4FC
LDL Subfractions, Free Cholesterol, LDL-5	L5FC
LDL Subfractions, Free Cholesterol, LDL-6	L6FC
LDL Subfractions, Phospholipids, LDL-1	L1PL
LDL Subfractions, Phospholipids, LDL-2	L2PL
LDL Subfractions, Phospholipids, LDL-3	L3PL
LDL Subfractions, Phospholipids, LDL-4	L4PL
LDL Subfractions, Phospholipids, LDL-5	L5PL
LDL Subfractions, Phospholipids, LDL-6	L6PL
LDL Subfractions, Triglycerides, LDL-1	L1TG
LDL Subfractions, Triglycerides, LDL-2	L2TG
LDL Subfractions, Triglycerides, LDL-3	L3TG
LDL Subfractions, Triglycerides, LDL-4	L4TG
LDL Subfractions, Triglycerides, LDL-5	L5TG
LDL Subfractions, Triglycerides, LDL-6	L6TG
Lipoprotein Main Fractions, Apo-A1, HDL	HDA1
Lipoprotein Main Fractions, Apo-A2, HDL	HDA2
Lipoprotein Main Fractions, Apo-B, IDL	IDAB
Lipoprotein Main Fractions, Apo-B, LDL	LDAB
Lipoprotein Main Fractions, Apo-B, VLDL	VLAB
Lipoprotein Main Fractions, Cholesterol, HDL	HDCH
Lipoprotein Main Fractions, Cholesterol, IDL	IDCH
Lipoprotein Main Fractions, Cholesterol, LDL	LDCH
Lipoprotein Main Fractions, Cholesterol, VLDL	VLCH
Lipoprotein Main Fractions, Free Cholesterol, HDL	HDFC
Lipoprotein Main Fractions, Free Cholesterol, IDL	IDFC
Lipoprotein Main Fractions, Free Cholesterol, LDL	LDFC
Lipoprotein Main Fractions, Free Cholesterol, VLDL	VLFC
Lipoprotein Main Fractions, Phospholipids, HDL	HDPL
Lipoprotein Main Fractions, Phospholipids, IDL	IDPL
Lipoprotein Main Fractions, Phospholipids, LDL	LDPL
Lipoprotein Main Fractions, Phospholipids, VLDL	VLPL
Lipoprotein Main Fractions, Triglycerides, HDL	HDTG
Lipoprotein Main Fractions, Triglycerides, IDL	IDTG
Lipoprotein Main Fractions, Triglycerides, LDL	LDTG
Lipoprotein Main Fractions, Triglycerides, VLDL	VLTG
Main Parameters, Apo-A1, Apo-A1	TPA1
Main Parameters, Apo-A2, Apo-A2	TPA2
Main Parameters, Apo-B100, Apo-B100	TPAB
Main Parameters, Cholesterol, Chol	TPCH
Main Parameters, HDL Cholesterol, HDL-Chol	HDCH
Main Parameters, LDL Cholesterol, LDL-Chol	LDCH
Main Parameters, Triglycerides, TG	TPTG
VLDL Subfractions, Cholesterol, VLDL-1	V1CH

VLDL Subfractions, Cholesterol, VLDL-2	V2CH
VLDL Subfractions, Cholesterol, VLDL-3	V3CH
VLDL Subfractions, Cholesterol, VLDL-4	V4CH
VLDL Subfractions, Cholesterol, VLDL-5	V5CH
VLDL Subfractions, Free Cholesterol, VLDL-1	V1FC
VLDL Subfractions, Free Cholesterol, VLDL-2	V2FC
VLDL Subfractions, Free Cholesterol, VLDL-3	V3FC
VLDL Subfractions, Free Cholesterol, VLDL-4	V4FC
VLDL Subfractions, Free Cholesterol, VLDL-5	V5FC
VLDL Subfractions, Phospholipids, VLDL-1	V1PL
VLDL Subfractions, Phospholipids, VLDL-2	V2PL
VLDL Subfractions, Phospholipids, VLDL-3	V3PL
VLDL Subfractions, Phospholipids, VLDL-4	V4PL
VLDL Subfractions, Phospholipids, VLDL-5	V5PL
VLDL Subfractions, Triglycerides, VLDL-1	V1TG
VLDL Subfractions, Triglycerides, VLDL-2	V2TG
VLDL Subfractions, Triglycerides, VLDL-3	V3TG
VLDL Subfractions, Triglycerides, VLDL-4	V4TG
VLDL Subfractions, Triglycerides, VLDL-5	V5TG
